# Supplementary figures and images for: Hypoxia-inducible factor prolyl hydroxylase domain (PHD) inhibition after contusive spinal cord injury does not improve locomotor recovery
Source: PLoS One. 2021 Apr 5;16(4):e0249591. doi: 10.1371/journal.pone.0249591 (PMC8021188; doi:10.1371/journal.pone.0249591)

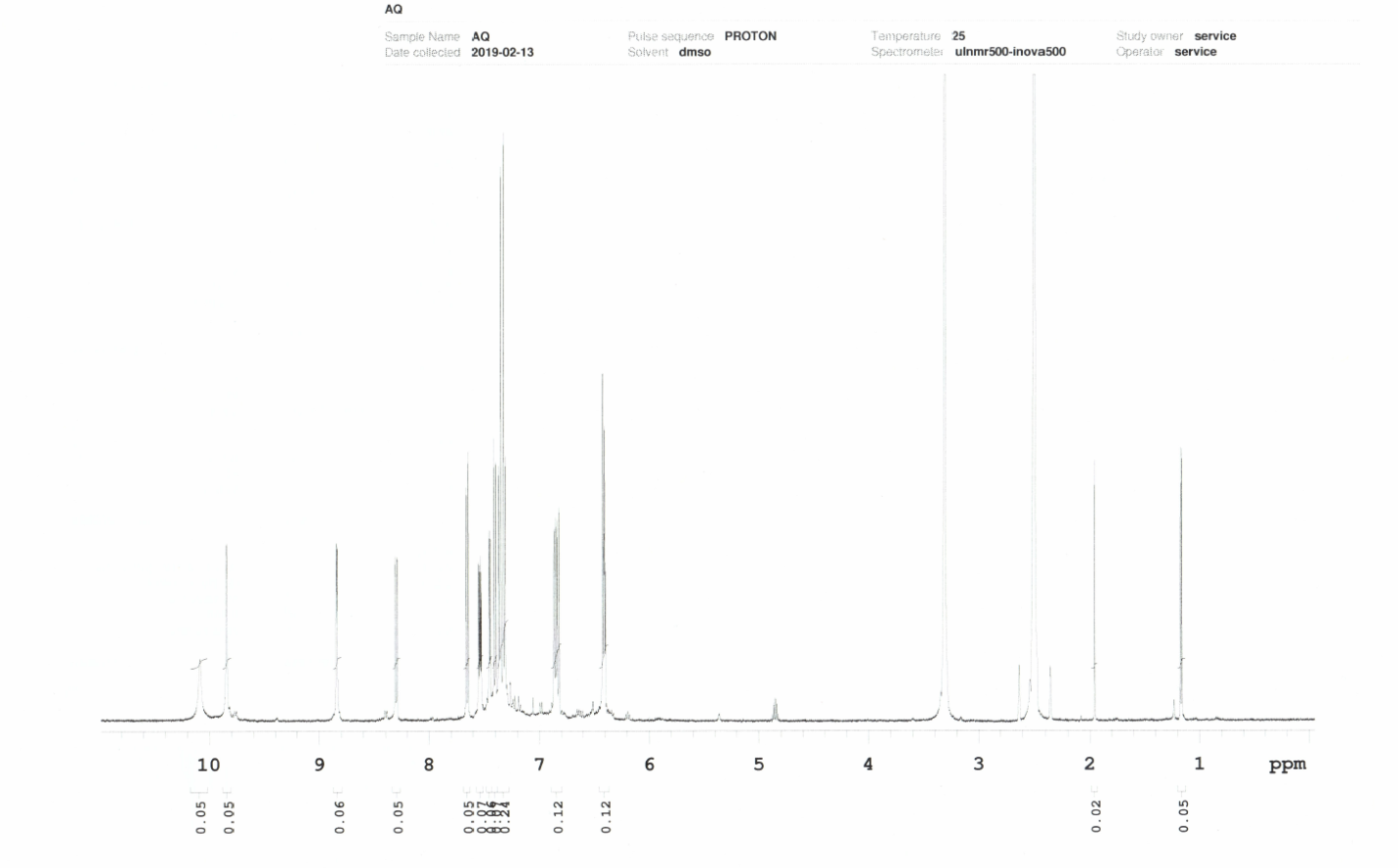

Supplement: S1 Fig — 1H NMR (DMSO-d6, 500 MHz) δ 9.84 (s, 1H), 8.84 (dd, 1H), 8.2–8.4 (m, 1H), 7.66 (d, 1H), 7.54 (dd, 1H), 7.3–7.5 (m, 6H), 6.8–6.9 (m, 2H), 6.4–6.5 (m, 2H) matches the spectra of AQ as previously described [56]. Data were recorded on a Varian Unity Inova 500 MHz and chemical shifts are reported in ppm using the solvent as an internal standard (DMSO-d6 at 2.5 ppm). (TIF) [file pone.0249591.s001.tif]

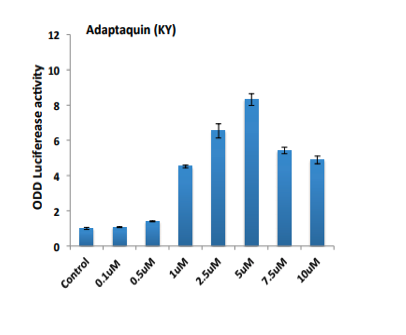

Supplement: S2 Fig — SH-SY5Y ODD-Luc cells were treated with increasing concentrations of AQ (0.1 μM-10 μM) for 3 h. ODD-luciferase activity was determined by luminometry. AQ inhibits HIF prolyl hydroxylase activity and stabilize luciferase fused to the oxygen degradation domain (ODD). (TIF) [file pone.0249591.s002.tif]

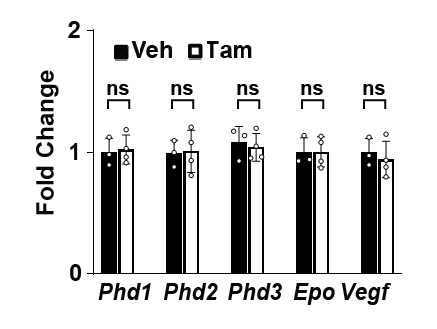

Supplement: S3 Fig — Tamoxifen (Tam)-treated Plp-creERT2+/+:Egln1/2/3fl/fl mice (n = 4, M:F = 2:2) show no changes in Phd1/Egln1, Phd2/Egln2, Phd3/Egln3, Epo, and Vegf mRNAs (controls received vehicle /Veh/, n = 3, M:F = 2:1). (TIF) [file pone.0249591.s003.tif]
