# Supplementary material for: Hypoxia-inducible factor prolyl hydroxylase domain (PHD) inhibition after contusive spinal cord injury does not improve locomotor recovery
Source: PLoS One. 2021 Apr 5;16(4):e0249591. doi: 10.1371/journal.pone.0249591 (PMC8021188; doi:10.1371/journal.pone.0249591)
Supplement: S1 Table — (DOCX) [file pone.0249591.s004.docx]

**S1 Table.** Experimental Design.

| **Study (figure panel)** | **Groups** | **N (M:F)** | **Number of animals lost to peri- or post-operative mortality (M:F)** |
| --- | --- | --- | --- |
| Analyzing effects of AQ on neural cell-specific and ISR/ERSR and ATF4-regulated gene mRNA levels after SCI (Fig 1a, b) | WT sham @ 72h  WT SCI Veh@72h  WT SCI AQ@72h | 8 (0:8)  8 (0:8)  8 (0:8) | None |
| Analyzing effects of AQ on locomotor recovery (Fig 1c) | WT SCI Veh  WT SCI AQ | 8 (0:6)  8 (0:7) | 2 (0:2)^b^  1 (0:1)^b^ |
| Analyzing effects AQ on WMS (Fig 1d, e) | WT SCI Veh@ dpi 42  WT SCI AQ@ dpi 42 | 4 (0:4)  4 (0:4) | None |
| Analysis of HIF-PHDs deletion on HIF-dependent gene mRNA levels (Fig 2a) | Spinal Cord  *Plp-cre^ERT2+/+^:* Egln *1,2,3^fl/fl^* Veh  *Plp-cre^ERT2+/+^:* Egln *1,2,3^fl/fl^* Tam  Optic Nerve  *Plp-cre^ERT2+/+^:* Egln *1,2,3^fl/fl^* Veh  *Plp-cre^ERT2+/+^:* Egln *1,2,3^fl/fl^* Tam | 9 (4:5) ^a^  10 (4:6) ^a^  3 (2:1) ^a^  4 (2:2) ^a^ | None |
| Analyzing effects of HIF-PHDs deletion on neural cell-specific and ISR/ERSR regulated gene mRNA levels after SCI (Fig 2b, c) | *Plp-cre^ERT2+/+^:* Egln1*,2,3^fl/fl^* sham@ 72h  *Plp-cre^ERT2+/+^:* Egln*1,2,3^fl/fl^* Veh SCI@ 72h  *Plp-cre^ERT2+/+^:* Egln*1,2,3^fl/fl^* Tam SCI@ 72h | 4 (2:2)  4 (2:2)  4 (2:2) | None |
| Analyzing effects of HIF-PHDs deletion on locomotor recovery (Fig 2d) | *Plp-cre^ERT2+/+^:* Egln*1,2,3^fl/fl^* Veh SCI  *Plp-cre^ERT2+/+^:* Egln*1,2,3^fl/fl^* Tam SCI  WT Tam SCI | 11 (5:6)  14 (7:7) ^a^  12 (6:6) ^a^ | 3 (3:0)^b^  2 (2:0)^b^  2 (2:0)^b^ |

^a^ unbalanced gender ratios were caused by limited availability of males of comparable age

^b^ in cases, when animals were lost before completion of all behavioral assessments, their individual scores were excluded from group analyses
