# Supplementary material for: Hypoxia-inducible factor prolyl hydroxylase domain (PHD) inhibition after contusive spinal cord injury does not improve locomotor recovery
Source: PLoS One. 2021 Apr 5;16(4):e0249591. doi: 10.1371/journal.pone.0249591 (PMC8021188; doi:10.1371/journal.pone.0249591)
Supplement: S2 Table — (DOCX) [file pone.0249591.s005.docx]

**S2 Table.** List of qPCR primers.

| Gene | Assay ID (ThermoFisher Scientific TaqMan Assay) |
| --- | --- |
| *Atf4* | Mm00515324_m1 |
| *Chop* | Mm01135937_g1 |
| *Gadd34* | Mm00492555_m1 |
| *Grp78* | Mm01333323_g1 |
| *Mbp* | Mm00521980_1 |
| *Olig2* | Mm01210556_m1 |
| *Nse* | Mm00468052 |
| *Map2* | Mm00485230_m1 |
| *Xbp* | Mm00457359_m1 |
| Gene | Primer sequences used for SYBR Green reactions |
| *Phd1* | For: 5’- ATGGCTCACGTGGACGCAGTAA-3’  Rev: 5’- CATTGCCTGGATAACACGCCAC-3’ |
| *Phd2* | For: 5’- TAAACGGCCGAACGAAAGC-3’  Rev: 5’- GGGTTATCAACGTGACGGACA-3’ |
| *Phd3* | For: 5’- CTATGTCAAGGAGCGGTCCAA-3’  Rev: 5’- GTCCACATGGCGAACATAACC-3’ |
| *Epo* | For: 5’- CATCTGCGACAGTCGAGTTCTG-3’  Rev: 5’- CACAACCCATCGTGACATTTTC-3’ |
| *Vegfa* | For: 5’- CTGCTGTAACGATGAAGCCCTG-3’  Rev: 5’- GCTGTAGGAAGCTCATCTCTCC-3’ |
