# Supplementary material for: Hypoxia-inducible factor prolyl hydroxylase domain (PHD) inhibition after contusive spinal cord injury does not improve locomotor recovery
Source: PLoS One. 2021 Apr 5;16(4):e0249591. doi: 10.1371/journal.pone.0249591 (PMC8021188; doi:10.1371/journal.pone.0249591)
Supplement: S3 Table — (DOCX) [file pone.0249591.s006.docx]

**S3 Table.** Raw BMS score data for individual mice.

| Genotype | Treatment | Mouse ID | baseline | dpi^a^ 3 | dpi^a^ 7 | dpi^a^ 14 | dpi^a^ 21 | dpi^a^ 28 | dpi^a^ 35 | dpi^a^ 42 |
| --- | --- | --- | --- | --- | --- | --- | --- | --- | --- | --- |
| C57BL/6 | Veh | 70822 | 9 | 2.5 | 4 | 4.5 | 5 | 5 | 5 | 5 |
| C57BL/6 | Veh | 70839 | 9 | 2 | 4 | 5 | 5 | 5 | 6 | 5 |
| C57BL/6 | Veh | 70848 | 9 | 3.5 | 4 | 5 | 5 | 5 | 6 | 5 |
| C57BL/6 | Veh | 70813 | 9 | 1.5 | 4 | 4 | 5 | 5 | 5 | 5 |
| C57BL/6 | Veh | 70868 | 9 | 1 | 4 | 4 | 4.5 | 5 | 5 | 4 |
| C57BL/6 | Veh | 70894 | 9 | 1 | 4 | 4 | 5 | 5 | 5 | 5 |
| C57BL/6 | AQ | 70860 | 9 | 2 | 5 | 4 | 5 | 6 | 5.5 | 5 |
| C57BL/6 | AQ | 70861 | 9 | 1.5 | 4 | 5 | 5 | 5 | 5 | 5 |
| C57BL/6 | AQ | 70883 | 9 | 2.5 | 4 | 4 | 5 | 5 | 5 | 5 |
| C57BL/6 | AQ | 70815 | 9 | 1 | 3 | 4 | 4 | 5 | 4 | 4.5 |
| C57BL/6 | AQ | 70883 | 9 | 1.5 | 4 | 5 | 5 | 5 | 5 | 5 |
| C57BL/6 | AQ | 70898 | 9 | 1.5 | 3.5 | 4 | 4 | 5 | 5 | 5 |
| C57BL/6 | AQ | 70899 | 9 | 3 | 4.5 | 5 | 5 | 5 | 5 | 5 |
| *Egln1/2/3^fl/fl^* : *Plp-Cre^ERT2^* | Tam | 30178 | 9 | 3 | 4 | 4 | 4.5 | 5 | 5 | 5 |
| *Egln1/2/3^fl/fl^* : *Plp-Cre^ERT2^* | Tam | 30153 | 9 | 4 | 4 | 5 | 5 | 5 | 5 | 5 |
| *Egln1/2/3^fl/fl^* : *Plp-Cre^ERT2^* | Tam | 30476 | 9 | 1.5 | 5 | 5 | 5 | 5 | 5 | 4 |
| *Egln1/2/3^fl/fl^* : *Plp-Cre^ERT2^* | Tam | 30268 | 9 | 1.5 | 4 | 4 | 4 | 5 | 5 | 5 |
| *Egln1/2/3^fl/fl^* : *Plp-Cre^ERT2^* | Tam | 30289 | 9 | 1 | 6 | 6 | 6 | 5 | 6 | 5 |
| *Egln1/2/3^fl/fl^* : *Plp-Cre^ERT2^* | Tam | 30252 | 9 | 2 | 3 | 4 | 4.5 | 5 | 5 | 5 |
| *Egln1/2/3^fl/fl^* : *Plp-Cre^ERT2^* | Tam | 30274 | 9 | 1.5 | 4 | 4 | 4.5 | 5 | 5 | 5 |
| *Egln1/2/3^fl/fl^* : *Plp-Cre^ERT2^* | Tam | 30492 | 9 | 2 | 4 | 5 | 5 | 5 | 5 | 5 |
| *Egln1/2/3^fl/fl^* : *Plp-Cre^ERT2^* | Tam | 30433 | 9 | 2.5 | 5 | 5 | 5 | 5 | 4.5 | 5 |
| *Egln1/2/3^fl/fl^* : *Plp-Cre^ERT2^* | Tam | 30254 | 9 | 1.5 | 4 | 4 | 4 | 4.5 | 5 | 5 |
| *Egln1/2/3^fl/fl^* : *Plp-Cre^ERT2^* | Tam | 30220 | 9 | 1.5 | 4 | 5 | 5 | 5 | 5 | 5 |
| *Egln1/2/3^fl/fl^* : *Plp-Cre^ERT2^* | Tam | 30349 | 9 | 2 | 4 | 5 | 5 | 5 | 5 | 5 |
| *Egln1/2/3^fl/fl^* : *Plp-Cre^ERT2^* | Veh | 30191 | 9 | 2.5 | 5 | 5 | 5 | 5 | 5 | 5 |
| *Egln1/2/3^fl/fl^* : *Plp-Cre^ERT2^* | Veh | 30437 | 9 | 2 | 4 | 4 | 5 | 4.5 | 5 | 6.5 |
| *Egln1/2/3^fl/fl^* : *Plp-Cre^ERT2^* | Veh | 30356 | 9 | 1.5 | 4 | 5 | 5 | 4.5 | 5 | 5 |
| *Egln1/2/3^fl/fl^* : *Plp-Cre^ERT2^* | Veh | 30145 | 9 | 2 | 4 | 3 | 5 | 5 | 5 | 5 |
| *Egln1/2/3^fl/fl^* : *Plp-Cre^ERT2^* | Veh | 30141 | 9 | 2.5 | 4 | 4 | 5 | 4.5 | 5 | 5 |
| *Egln1/2/3^fl/fl^* : *Plp-Cre^ERT2^* | Veh | 30456 | 9 | 1.5 | 2.5 | 5 | 5 | 5 | 5 | 5 |
| *Egln1/2/3^fl/fl^* : *Plp-Cre^ERT2^* | Veh | 30130 | 9 | 2 | 4 | 4 | 5 | 5.5 | 5 | 5 |
| *Egln1/2/3^fl/fl^* : *Plp-Cre^ERT2^* | Veh | 30146 | 9 | 3 | 3.5 | 5 | 5 | 5 | 5 | 5 |
| C57BL/6 | Tam | 30464 | 9 | 1 | 4 | 4 | 4.5 | 5 | 5 | 5 |
| C57BL/6 | Tam | 30184 | 9 | 2 | 4 | 4 | 4 | 5 | 4 | 4.5 |
| C57BL/6 | Tam | 30149 | 9 | 2 | 4.5 | 4 | 5 | 5 | 5 | 5 |
| C57BL/6 | Tam | 30446 | 9 | 2.5 | 4 | 4 | 5 | 5 | 5 | 4 |
| C57BL/6 | Tam | 30471 | 9 | 2 | 4 | 4 | 4.5 | 5 | 5 | 5 |
| C57BL/6 | Tam | 30345 | 9 | 3 | 4 | 5 | 5 | 5 | 5 | 5 |
| C57BL/6 | Tam | 30299 | 9 | 3 | 3.5 | 4 | 5 | 5 | 5 | 5.5 |
| C57BL/6 | Tam | 30299 | 9 | 2.5 | 4 | 5 | 4 | 5 | 5 | 5 |
| C57BL/6 | Tam | 30173 | 9 | 2.5 | 4 | 4 | 5 | 5 | 5 | 5 |
| C57BL/6 | Tam | 30478 | 9 | 2.5 | 4 | 4 | 5 | 5 | 5 | 4.5 |

^a^ day post injury
